# Pharmacokinetics of Dantrolene in the Plasma Exchange Treatment of Malignant Hyperthermia in a 14-Year-Old Chinese Boy: A Case Report and Literature Review

**DOI:** 10.3389/fmed.2022.918245

**Published:** 2022-08-12

**Authors:** Xiaoxiao Li, Chao Li, Yang Zhou, Zhengqian Li, Xin Xiong, Chuhui Wang, Congya Zhou, Bin Han, Li Yang, Xiangyang Guo

**Affiliations:** ^1^Department of Pharmacy, Peking University Third Hospital, Beijing, China; ^2^Department of Pharmacy Administration and Clinical Pharmacy, School of Pharmaceutical Sciences, Peking University, Beijing, China; ^3^Department of Critical Care, Peking University Third Hospital, Beijing, China; ^4^Department of Anesthesiology, Peking University Third Hospital, Beijing, China; ^5^Beijing Center of Quality Control and Improvement on Clinical Anesthesia, Beijing, China

**Keywords:** malignant hyperthermia, dantrolene, pharmacokinetics, plasma exchange, general anesthesia

## Abstract

Malignant hyperthermia (MH) is a rare life-threatening response that is triggered by exposure to specific anesthetics commonly used during surgical interventions. Dantrolene is a well-known drug used as the first-line therapy for MH. A 14-year-old Chinese boy with a mutation in type 1 Ryanodine receptor (RyR1) whose muscle biopsy diagnosis was central core disease (CCD) had an occurrence of MH after a cervical spine surgery, during which he was placed under general anesthesia without volatile anesthetics or succinylcholine. The MH crisis treatment workflow was started and intravenous dantrolene was used, which was soon combined with sequent continuous veno-venous hemofiltration (CVVH) and plasma exchange (PE) therapy. We explored the pharmacokinetic profile of dantrolene during PE treatment. It showed that a one-compartment model with first-order kinetics was sufficient to characterize dantrolene pharmacokinetics (PK). The renal clearance estimate for dantrolene was 0.33 mL/(min*kg) and the volume of distribution was 0.51 L/kg. Though a 4-h PE elevated about 27% off-clearance for dantrolene, it eliminated extra dantrolene by a mere 4% of the area under the curve (AUC). We made no recommendation with respect to adjusting dantrolene dosing for MH adolescents with a 4-h PE.

## Introduction

Malignant hyperthermia (MH) is a rare life-threatening response that occurs in genetically susceptible individuals triggered by exposure to halogenated volatile anesthetics commonly used during surgery interventions, characterized by muscle rigidity, hyperthermia, rhabdomyolysis, acidosis, tachycardia, and, if untreated, death (1). It has probably occurred in 1:100,000 general anesthetics, but the prevalence rate is subject to underestimates as the lack of large sample investigation and the fact that many cases were not reported (2). Dantrolene is a selective inhibitor of type 1 Ryanodine receptor (RyR1) that suppresses Ca^2+^ release from the sarcoplasmic reticulum in skeletal muscle and is used as a therapeutic agent in individuals susceptible to MH (3). However, few pharmacokinetic data on dantrolene were reported when dantrolene was used in combination with blood purification, especially in MH children and adolescents. Whether blood purification affects the pharmacokinetic process of dantrolene remains unknown. Here, we report a 14-year-old Chinese boy unexposed to triggering anesthetics but occurred fulminant MH after cervical spine surgery. He was successfully cured by combined treatment of intravenous dantrolene, continuous veno-venous hemofiltration (CVVH), and plasma exchange (PE) therapy. In this case, the pharmacokinetics (PK) of intravenous dantrolene with PE during the MH crisis was explored.

## Case Presentation

**FIGURE 1:**
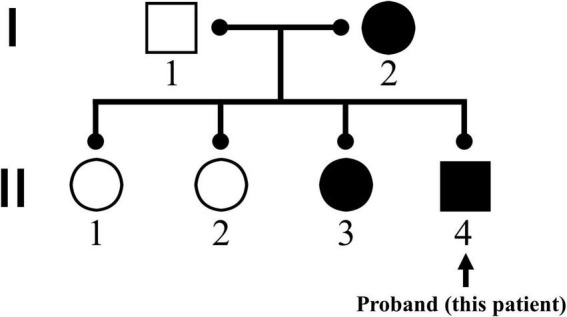
Family genogram for *RYR1* (NM 001042723) mutations. The patient’s mother and third sister were confirmed to have homologous gene mutations, while his father as well as eldest sister and second sister did not find the same mutations in genetic testing.

A 14-year-old boy with a 7-year history of progressively worsening cervical lordosis deformity was hospitalized and planned for elective cervical spine surgery on November 13, 2018. He suffered from progressive muscle stiffness in the posterior side of the neck and chest 7 years ago and developed shortness of breath and dysphagia 10 months ago. In combination with pathological results of muscle biopsy, he was diagnosed with the central core disease (CCD). Subsequent genetic testing revealed mutations on *RYR1* (NM 001042723) c.11120A > G and c.12227C > T (Figure 1).

This 42-kg-weight patient was afebrile with normal vital signs, while his preoperative laboratory tests, electrocardiograph (ECG), and Chest X-ray were within normal limits. He later underwent posterior cervical C3-T3 soft tissue release, pedical screw internal fixation and fusion, and excessive lordosis deformity. One day before the operation, the volatile tank of the anesthetic machine was removed, the sodium lime and breathing loop were replaced, and we flushed the loop using fresh gas 10 L/min for 10 min. Owing to his *RYR1* mutations, propofol 100 mg, sufentanil 20 μg and rocuronium 30 mg were intravenous injected for induction and the anesthesia was maintained with propofol and remifentanil throughout the operation. Neither volatile anesthetic agents nor succinylcholine was administered during the uneventful surgical procedure. The surgery lasted for 474 min and the total duration of anesthesia was 621 min.

The intraoperative plasma creatine phosphokinase (CK), creatine kinase myocardial band (CK-MB), and myoglobin were all within the normal range. The patient was transferred to the intensive care unit (ICU) after the surgery for further observation.

The patient was stable at ICU admission: BP 110/70 mmHg, HR 50∼60 bpm, SpO_2_ 100%, axillary temperature 35.0°C. Analgesia-sedation during the invasive mechanical ventilation was maintained with propofol (1∼1.5 mg/kg) and butorphanol (0.2∼0.25 mg/h). Ninety minutes after ICU admission, he developed a sudden onset sinus tachycardia of 140∼160 bpm while arterial blood pressure was 120∼130/70∼80 mmHg. At that moment, masseter muscle rigidity (MMR) appeared and muscle rigidity intermittently generalized, accompanied by an increase in axillary temperature from 36.0 to 39.4°C. Arterial blood gases showed mixed metabolic and respiratory acidosis with hyperkalemia (pH 7.236, PaCO_2_ 52.3 mmHg, K^+^ 4.38 mmol/L, Lac 5.7 mmol/L). A clinical diagnosis of fulminant MH was made according to the clinical grading scale (CGS) and scored 63 points (4).

The MH crisis treatment workflow was started immediately and dantrolene sodium (NDC 42023-123-06, Par Pharmaceutical Companies, Inc., United States, LOT 301039) was administered on the second day at an initial dose of 2.5 mg/kg followed by a maintenance dose of 0.25 mg/(kg*h) as the drugs were transformed from other hospitals (Supplementary Table 1). Due to the limited number of agents, usage of dantrolene was interrupted on the third night and the second bolus of dantrolene 1 mg/kg was administered on the fourth day. For maximizing ventilation and lowering the PaCO_2_, we raised the inspired oxygen fraction to 100%, accelerated respiratory frequency, and enhanced tidal volume immediately. Hypothermia therapy was conducted with a water-circulating cooling blanket as well as an ice cap. Then PaCO_2_ reduced to 28.7 mmHg and the axillary temperature dropped to 38.1°C. However, the status of the patient deteriorated over the next few hours, presenting as an observed intermittently MMR, along with a progressive increase of CK, serum myoglobin (Mb), urine myoglobin (UMb), and lactate dehydrogenase (LDH). Maximal plasma concentrations of CK and Mb were, respectively 3,303 IU/L and 579 ng/mL. Accordingly, a diagnosis of rhabdomyolysis syndrome was ascertained. Bedside CVVH (AQUARIUS, Nikkiso Europe, Langenhagen, Germany) was then initiated at 17 h after MH occurred, the hemofilter (HF1200) was a 1.25 m^2^ glycerin-free polysulfone-membrane (Polyflux, Medivators, Minneapolis, MN, United States). The replacement solution was a bicarbonate-based solution containing 1.91 mg/mL glucose and 0.02 mg/ml magnesium, while the predilution and postdilution rates were both 1,000 mL/h. Blood flow and fluid loss rates were 180 mL/min and 200 mL/h, respectively. The rectification of acid intoxication and urine alkalization, as well as several supportive treatments were performed at the same time. The values of CK, Mb, and LDH decreased slowly after administering CVVH for nearly 36 h, while his muscle tremors still occurred intermittently. Due to the poor effect of CVVH, we decided to treat his rhabdomyolysis syndrome with PE. The plasma filter was a 0.6 m^2^ polyurethane-membrane (Polyflux; Fresenius, Bad Homburg, Germany). Plasma rate and total volumes were 1,000 mL/min and 3000 ml, respectively.

Artery blood samples were collected before and after PE, followed by those collected after dantrolene administration for up to 12 h (at minutes 0, 15, 30, 60, 120, 240, 360 for the first bolus as 2.5 mg/kg dantrolene, and at minutes 0, 30, 120, 240, 360 for the second bolus as 1 mg/kg dantrolene). To minimize the harm of sampling, only 1 mL artery blood was collected from the boy at each time point. Plasma was separated by centrifuge and stored at −80°C until assayed.

The plasma concentrations of dantrolene were measured simultaneously by using a validated high-performance liquid chromatography (Shimadzu LC-30A; Shimadzu, JP) coupled with tandem mass spectrometry (ABSciex 4000^+^ triple quadrupole system, ABSciex Corp., United States) assay method. Data were acquired and processed by Analyst 1.6.2 software (ABSciex Corp). A 50 μL plasma sample was extracted by protein precipitation.

**FIGURE 2:**
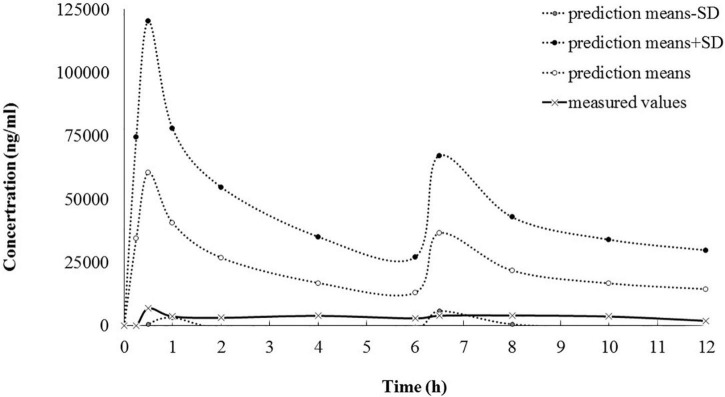
The goodness of fit between predicted and measured dantrolene plasma concentration (ng/ml). According to the Malignant Hyperthermia Association of the United States (MHAUS) guidelines (2.5 mg/kg as the first bolus and followed by 1 mg/kg bolus every 6 h), predicted concentrations of dantrolene were calculated with the reported population PK model (5) and didn’t match well with the measured concentrations over the entire concertation range after PE. PE, plasma exchange; PK, pharmacokinetics.

A reported population PK model, with the parameters from healthy adults, was applied for predicting dantrolene plasma concentrations (5). For all calculations, NONMEM software (version 7.3.0, ICON Development Solutions) was used. However, the predictions matched with the measured values only nearby the lower limit, suggesting new pharmacokinetic parameters for dantrolene should be estimated, which might be more suitable for MH adolescents (Figure 2). Thus, PK analysis was performed by using a non-linear mixed-effects model. Data were analyzed using a first-order conditional estimation method (6). One- and two-compartment models with first-order kinetics were investigated to determine the optimal structural model. The clearance (CL), volume of distribution (Vd), and area under the curve (AUC) of dantrolene were characterized and estimated. Because there was only one MH patient, the inter-individual variability was fixed as 0 and no covariate was retained in the final model.

**FIGURE 3:**
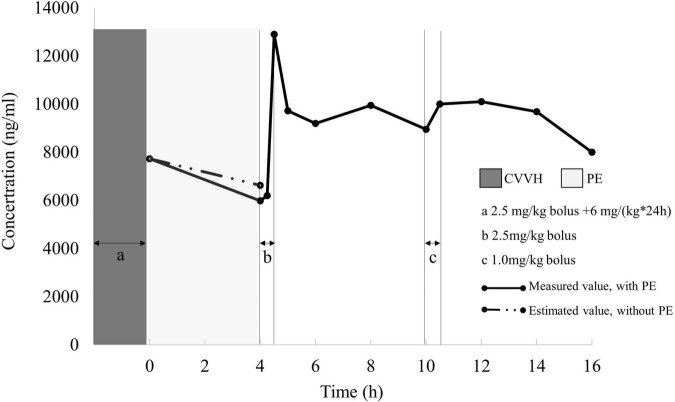
Measured dantrolene plasma concentration (dots) during PE and renal clearance period. Under the hypotheses of invariable renal function, pharmacokinetic parameters calculated from 4 to 16 h (after PE, only with renal clearance) was applied to stimulate AUC in the first 4 h for dantrolene. The eliminated dantrolene during the first 4 h was 27,420 ng*h/mL with PE, while decreased to 27,302 ng*h/mL if without PE. PE, plasma exchange; AUC, area under the curve.

A one-compartment model with first-order kinetics was sufficient to characterize dantrolene pharmacokinetics. The renal CL estimate for dantrolene was 0.33 mL/(min*kg) and Vd was 0.51 L/kg, the details were shown in Table 1 and Figure 3. His estimated CL elevated about 27% if combined with PE. However, the 4-h PE increased the eliminated dantrolene by a mere 4% of AUC.

**TABLE 1:** Pharmacokinetic parameters estimated for dantrolene.

Period	CL, mL/(min*kg)	V, L/kg
(A)	0.42	0.38
(B)	0.33	0.51

*(A) During PE period, (B) after PE, only with renal clearance.*

## Discussion

Malignant hyperthermia is a rare but potentially lethal disorder that is triggered by volatile anesthetics and depolarizing muscle relaxants. Most of the patients who suffered MH were reported to have a genetic defect in the *RYR1*, which was associated with disordered calcium channel function and disease. This adolescent patient was diagnosed as CCD with *RYR1* mutations and occurred MH postoperatively even absence of volatile anesthetics or depolarizing muscle relaxants. Dantrolene is the only clinically available agent for the specific MH treatment and is proven to decrease the mortality of MH from 70 (7) to <10% (3). However, how to adjust the dosage of dantrolene during blood purification remains unknown. To answer the question, we conducted a pilot study from a pharmacokinetic view on an MH adolescent receiving PE.

As an orphan drug, studies focused on PK parameters of dantrolene were limited worldwide. Besides, a gap existed between the enrolled volunteers/patients and this patient both in the demographic characteristics and physical status, making the generalizability border of available data vague. There was only one population PK model reported previously, by assuming one single compartment, the parameters were from 27 ± 5 yr old, weighted 70 ± 12 kg healthy adults (5). However, compared with the measured ones, the simulated dantrolene plasma concentrations for our MH adolescent patient (14-year-old, 48 kg) showed poor goodness of fit while applying the previous population PK model (healthy adults) (5). Also, the parameters using the non-compartmental method from another study were calculated as MH susceptible children during anesthesia aged 3.6 ± 1.3 years old and weighted 17.4 ± 4.9 kg (8). Due to the absence of key parameters in this published single dose model (8), we failed in predicting dantrolene plasma concentrations. However, the Vd calculated from our case (0.51 L/kg) was similar to that estimated in the previous children’s model (0.54 L/kg) (8).

In addition, other PK parameters, i.e., CL only based on renal clearance, for dantrolene administration in this adolescent patient presented as 0.33 mL/(min*kg). It was lower compared to those reported previously both in healthy adults 0.43 ± 0.043 mL/(min*kg) (5) and in MH susceptible children 0.64 ± 0.18 mL/(min*kg) (8). The difference might be caused by two reasons: (1) because of the high Mb concentrations, an acute kidney injury during the MH attack might occur, followed by a decreased elimination of dantrolene, and (2) a coefficient of variation might lie in the assay methods for the measurement of dantrolene among different studies. High-performance liquid chromatography was used to measure the whole blood (8) or plasma concentration of dantrolene (5) in previous studies, while high-performance liquid chromatography coupled with tandem mass spectrometry was applied to measure plasma level of dantrolene in this case.

Owing to the massive rhabdomyolysis complicated with MH, initiation of PE in early stage seems important (9). However, until now, there was no recommendation about how to adjust the dosage of dantrolene with PE treatment. This case is the first one to explore the pharmacokinetic profile of dantrolene involved in PE. Although CL increased from 0.33 to 0.42 mL/(min*kg) by a 27% uptrend with PE, the extra dantrolene eliminated by PE was only 4% in AUC. Considering the safety and cost-effectiveness, together with the accumulation of dantrolene, we made no recommendation concerning adjusting dantrolene dosing for MH adolescents with a 4-h PE. In addition, a loading dose of 2.5 mg/kg dantrolene after PE was unnecessary.

## Conclusion

The renal clearance estimate for dantrolene was 0.33 mL/(min*kg) and the volume of distribution was 0.51 L/kg. We made no recommendation with respect to adjusting dantrolene dosing for MH adolescents with a 4-h PE.

## Data Availability Statement

The original contributions presented in this study are included in the article/Supplementary Material, further inquiries can be directed to the corresponding authors.

## Ethics Statement

Ethical review and approval was not required for the study on human participants in accordance with the local legislation and institutional requirements. Written informed consent was obtained from the minor(s)’ legal guardian/next of kin for the publication of any potentially identifiable images or data included in this article.

## Author Contributions

XL was responsible for designing sampling strategies, collecting samples, calculating PK parameters, and drafting the manuscript. CL was responsible for the treatment decision, collecting clinical data, and involved in drafting the manuscript. YZ, ZL, and BH were responsible for offering clinical data and revising the manuscript. XX was responsible for establishing a measuring method of plasma concentrations. CW was involved in calculating PK parameters and revising the manuscript. CZ was responsible for the plasma concentrations of dantrolene determination. LY was responsible for the access to dantrolene, checking sampling strategies and measured data, and approving the final version of the manuscript. XG was responsible for the treatment decision, the access to dantrolene, getting informed consent from parents, and approving the final version of the manuscript. All authors contributed to the article and approved the submitted version.

## Conflict of Interest

The authors declare that the research was conducted in the absence of any commercial or financial relationships that could be construed as a potential conflict of interest.

## Publisher’s Note

All claims expressed in this article are solely those of the authors and do not necessarily represent those of their affiliated organizations, or those of the publisher, the editors and the reviewers. Any product that may be evaluated in this article, or claim that may be made by its manufacturer, is not guaranteed or endorsed by the publisher.
