# Supplementary material for: Pharmacokinetics of Dantrolene in the Plasma Exchange Treatment of Malignant Hyperthermia in a 14-Year-Old Chinese Boy: A Case Report and Literature Review
Source: Front Med (Lausanne). 2022 Aug 12;9:918245. doi: 10.3389/fmed.2022.918245 (PMC9411638; doi:10.3389/fmed.2022.918245)
Supplement: Supplementary file 1 [file Table_1.docx]

**Supplement table 1 The important monitoring items during malignant hyperthermia crisis treatment**

| **Time** | **CK**  **(IU/L)** | **CK-MB (IU/L)** | **LDH**  **(IU/L)** | **Mb**  **(ng/mL)** | **UMb (ng/mL)** | **DMb**  **(ng/mL)** | **K^+^**  **(mmol/L)** | **Mg^2+^**  **(mmol/L)** | **Scr**  **(μmol/L)** | **BUN**  **(mmol/L)** | **USG** | **pH**  **(Urine)** | **pH**  **(Serum)** | **Lac**  **(mmol/L)** | **PaCO_2_**  **(mmHg)** | **T**  **(℃)** | **Dantrolene** | **CBP** |
| --- | --- | --- | --- | --- | --- | --- | --- | --- | --- | --- | --- | --- | --- | --- | --- | --- | --- | --- |
| **Baseline** | 204 | 18 |  | ＜21 |  |  | 3.96 |  | 63 | 5.0 | 1.039 | 5.5 |  |  |  |  |  |  |
| 11-13 | | | | | | | | | | | | | | | | | | |
| 18:00 | 263 | 19 | 190 |  |  |  | 3.34 | 0.74 | 48 | 4.2 |  |  | 7.541 | 1.8 | 26.2 | ＜35 |  |  |
| 20:00 | 653 | 26 | 209 | 643.3 | 25.4 |  |  |  |  |  | 1.045 | 8.0 | 7.236 | 5.7 | 52.3 | 39.4 |  |  |
| 23:00 | 1155 | 30 | 239 | 475.7 | 33.8 |  | 3.47 | 0.72 |  |  | 1.037 | 8.0 | 7.467 | 1.7 | 28.7 | 37.9 |  |  |
| 11-14 | | | | | | | | | | | | | | | | | | |
| 1:20 | 1443 | 40 | 243 | 537.7 | 38.2 |  | 3.75 | 0.69 |  |  | 1.042 | 6.0 |  |  |  | 37.8 |  |  |
| 5:00 | 2458 | 56 | 247 | 575 | 147 |  | 3.64 |  | 47 | 3.6 | 1.033 | 7.5 | 7.417 | 1.8 | 38.6 | 38.3 |  |  |
| 11:00 | 3179 | 79 | 245 | 497.9 | 34.8 |  | 3.7 | 0.65 |  |  | 1.034 | 8.5 | 7.487 | 1.9 | 30.3 | 37.9 | 120 mg |  |
| 13:20 | 3268 | 85 | 264 | 391.7 | 21.3 |  | 3.88 |  |  |  |  |  |  |  |  | 37.9 | 12 mg/hr | CVVH |
| 15:38 | 3303 | 86 | 245 | 328.4 | ＜21 |  | 3.7 |  |  |  |  |  | 7.439 | 1.3 | 29.8 | 37.8 | ↓ | ↓ |
| 17:30 | 2284 | 90 | 232 | 282.7 | ＜21 |  | 3.8 |  |  |  | 1.018 | 6.0 | 7.453 | 1.2 | 29.8 | 37.4 | ↓ | ↓ |
| 19:25 | 1772 | 89 | 231 | 263.8 | ＜21 |  | 3.9 |  |  |  | 1.009 | 7.0 | 7.458 | 1.2 | 28.7 | 36.8 | ↓ | ↓ |
| 21:28 | 1167 | 92 | 232 | 226.5 | ＜21 |  | 4.2 |  |  |  | 1.021 | 5.5 | 7.325 | 1.3 | 38.6 | 36.5 | ↓ | ↓ |
| 23:20 | 1670 | 94 | 236 | 190.1 | ＜21 | 39.1 | 4.37 |  |  |  | 1.008 | 5.0 | 7.381 | 1.3 | 32.7 | 36.5 | ↓ | ↓ |
| 11-15 | | | | | | | | | | | | | | | | | | |
| 1:30 | 2190 | 88 | 220 | 159.5 | ＜21 | 32.7 | 4.3 |  |  |  | 1.015 | 5.5 |  |  |  | 36.5 | ↓ | ↓ |
| 3:30 | 1963 | 89 | 231 | 168.8 | ＜21 | 34.6 | 4.33 |  |  |  | 1.022 | 7.0 |  |  |  | 36.2 | ↓ | ↓ |
| 5:17 | 764 | 87 | 222 | 136.4 | ＜21 |  | 4.1 |  | 39 | 1.0 | 1.026 | 6.5 | 7.392 | 1.2 | 33.9 | 36.5 | ↓ | ↓ |
| 7:22 | 2213 | 80 | 222 | 117.5 | ＜21 | 23.6 | 4.26 |  |  |  | 1.013 | 6.5 |  |  |  | 36.5 | ↓ | ↓ |
| 12:00 | 2907 | 91 | 216 | 761.5 | ＜21 |  | 4.0 |  |  |  | 1.020 | 5.5 | 7.429 | 1.1 | 27.3 | 36.5 | ↓ | ↓ |
| 18:30 | 2143 | 97 | 224 | 543.3 | ＜21 |  | 3.95 |  |  |  | 1.023 | 8.0 | 7.462 | 1.1 | 25.6 | 37.3 | ↓ | ↓ |
| 23:00 | 3051 | 99 | 239 | 373.9 | ＜21 | 69.5 |  |  |  |  | 1.010 | 7.5 | 7.433 | 1.1 | 34 | 37.0 | ↓ | ↓ |

| **Time** | **CK**  **(IU/L)** | **CK-MB**  **(IU/L)** | **LDH**  **(IU/L)** | **Mb**  **(ng/mL)** | **UMb**  **(ng/mL)** | **DMb**  **(ng/mL)** | **K^+^**  **(mmol/L)** | **Mg^2+^**  **(mmol/L)** | **Scr**  **(μmol/L)** | **BUN**  **(mmol/L)** | **USG** | **pH**  **(Urine)** | **pH**  **(Serum)** | **Lac**  **(mmol/L)** | **PaCO_2_**  **(mmHg)** | **T**  **(℃)** | **Dantrolene** | **CBP** |
| --- | --- | --- | --- | --- | --- | --- | --- | --- | --- | --- | --- | --- | --- | --- | --- | --- | --- | --- |
| 11-16 0:30-4:30 **PE** | | | | | | | | | | | | | | | | | | |
| 4:30 |  |  |  | 252.9 | ＜21 | 270.6 |  | 0.72 |  |  |  |  |  |  |  |  |  |  |
| 5:30 |  |  |  |  |  |  |  |  |  |  |  |  |  |  |  |  | 120 mg |  |
| 6:00 | 1259 | 43 | 168 | 371.5 | ＜21 |  | 3.11 | 1.47 | 51 | 1.6 | 1.013 | 8.0 | 7.501 | 1.2 | 33.7 | 37.9 |  |  |
| 11:30 |  |  |  |  |  |  |  |  |  |  |  |  |  |  |  |  | 48 mg |  |
| 15:39 |  |  |  | 469.4 | ＜21 |  |  | 1.57 |  |  |  |  | 7.470 | 1.5 | 32.9 | 37.7 |  |  |
| 17:30 |  |  |  |  |  |  |  |  |  |  |  |  |  |  |  |  | 48 mg |  |
| 18:40 | 2694 | 66 | 195 |  |  |  |  |  |  |  |  |  | 7.476 | 1.5 | 29.4 | 37.7 |  |  |
| 21:00 | 2609 | 64 | 205 | 260.6 |  |  |  | 1.53 |  |  |  |  | 7.509 | 1.3 | 29.5 | 37.3 |  |  |
| 23:00 |  |  |  |  |  |  |  |  |  |  |  |  |  |  |  |  | 48 mg |  |
| 11-17 | | | | | | | | | | | | | | | | | | |
| 1:00 |  |  |  |  |  |  |  |  |  |  |  |  |  |  |  |  |  | CVVH |
| 5:00 |  |  |  |  |  |  | 3.79 |  | 47 | 3.8 |  |  | 7.456 | 1.1 | 30.7 | 37.6 | 48 mg | ↓ |
| 9:00 | 1572 | 78 | 220 | 207.4 |  | 40.1 |  | 1.22 |  |  | 1.028 | 8.5 | 7.417 | 1.1 | 38.6 | 37.3 |  | ↓ |
| 11:00 |  |  |  |  |  |  |  |  |  |  |  |  |  |  |  |  | 48 mg | ↓ |
| 15:00 | 2580 | 86 | 241 | 188.7 | ＜21 | 40.2 |  |  |  |  |  |  |  |  |  | 37.4 |  |  |
| 19:16 | 3065 | 103 | 262 | 109.1 | ＜21 |  | 4.28 |  |  |  | 1.023 | 8.0 | 7.403 | 1.1 | 38.3 | 37.0 |  |  |
| 11-18 | | | | | | | | | | | | | | | | | | |
| 0:00 |  |  |  |  |  |  |  |  |  |  |  |  |  |  |  |  | 120 mg |  |
| 3:00 | 2890 | 84 | 273 | 49 | ＜21 |  |  | 0.82 |  |  | 1.027 | 8.5 |  |  |  | 38.3 |  |  |
| 7:30 | 2468 | 71 | 250 | 156.9 | ＜21 |  | 3.78 |  | 40 | 2.9 | 1.026 | 8.5 | 7.492 | 1.2 | 31.9 | 37.8 |  |  |
| 13:30 | 3143 | 82 | 289 | 96.7 |  |  |  |  |  |  |  |  | 7.399 | 1.0 | 51.7 | 37.0 |  |  |
| 21:30 | 2615 | 60 | 939 | 61.3 |  |  |  |  |  |  |  |  | 7.388 | 1.2 | 52 | 37.2 |  |  |
| 11-19 | | | | | | | | | | | | | | | | | | |
| 5:00 | 1513 | 58 | 279 | 136.4 |  |  | 2.79 |  | 33 | 4.6 |  |  | 7.508 | 1.3 | 33.8 | 36.5 |  |  |

**Abbreviation**: **BUN**, blood urea nitrogen; **CBP**, [continuous](javascript:;) [blood](javascript:;) [purification](javascript:;); **CK-MB**, creatine kinase myocardial band; **CK**, creatine phosphokinase; **DMb**, myoglobin in discarded liquor generated from [continuous](javascript:;) [blood](javascript:;) [purification](javascript:;); **Lac**, lactic acid; **LDH**, lactate dehydrogenase; **Mb**, serum myoglobin; **PE**, plasma exchange; **T**, temperature; **UMb**, urine myoglobin; **USG**, urine specific gravity; **Scr**, serum creatinine.
